# An Ecological Study on the Spatially Varying Relationship between County-Level Suicide Rates and Altitude in the United States

**DOI:** 10.3390/ijerph15040671

**Published:** 2018-04-04

**Authors:** Hoehun Ha, Wei Tu

**Affiliations:** 1 Department of Sociology, Anthropology and Geography, Auburn University at Montgomery, 7041 Senators Drive, Montgomery, AL 36117, USA; 2 Department of Geology and Geography, Georgia Southern University, P.O. Box 8149, Statesboro, GA 30460, USA; wtu@georgiasouthern.edu

**Keywords:** altitude, suicide rates, geographically weighted regression, health geography

## Abstract

Suicide is a serious but preventable public health issue. Several previous studies have revealed a positive association between altitude and suicide rates at the county level in the contiguous United States. We assessed the association between suicide rates and altitude using a cross-county ecological study design. Data on suicide rates were obtained from a Web-based Injury Statistics Query and Reporting System (WISQARS), maintained by the U.S. National Center for Injury Prevention and Control (NCIPC). Altitude data were collected from the United States Geological Survey (USGS). We employed an ordinary least square (OLS) regression to model the association between altitude and suicide rates in 3064 counties in the contiguous U.S. We conducted a geographically weighted regression (GWR) to examine the spatially varying relationship between suicide rates and altitude after controlling for several well-established covariates. A significant positive association between altitude and suicide rates (average county rates between 2008 and 2014) was found in the dataset in the OLS model (*R*^2^ = 0.483, *p* < 0.001). Our GWR model fitted the data better, as indicated by an improved *R*^2^ (average: 0.62; range: 0.21–0.64) and a lower Akaike Information Criteria (AIC) value (13,593.68 vs. 14,432.14 in the OLS model). The GWR model also significantly reduced the spatial autocorrelation, as indicated by Moran’s I test statistic (Moran’s *I* = 0.171; z = 33.656; *p* < 0.001 vs. Moran’s *I* = 0.323; z = 63.526; *p* < 0.001 in the OLS model). In addition, a stronger positive relationship was detected in areas of the northern regions, northern plain regions, and southeastern regions in the U.S. Our study confirmed a varying overall positive relationship between altitude and suicide. Future research may consider controlling more predictor variables in regression models, such as firearm ownership, religion, and access to mental health services.

## 1. Introduction

Suicide, increasingly recognized as a serious but preventable public health problem, is an important cause of global mortality [1,2]. Suicide claimed close to 800,000 lives worldwide in 2015, accounting for 1.4% of the total of all-cause deaths [3]. In the U.S. in 2015, there were more than 44,000 reported suicide deaths, making it the 2nd and 10th leading cause of death for the population aged between 15 and 34 and for all age groups, respectively. In addition, 9.8 million adults aged 18 or older had serious suicidal thoughts, and 1.4 million attempted suicide. The economic burden of suicide in 2013 alone was estimated to be between US$50.8 billion [4] and $93.5 billion [5]. Moreover, suicide rates increased from 10.5 per 100,000 in 1999 to 13.0 per 100,000 in 2015, a 24% growth in less than 20 years [6].

Past studies have implicated several risk factors for suicide, including demographic (e.g., certain age cohorts, white males, marital status), socioeconomic (e.g., poverty, unemployment, social isolation), environmental (e.g., altitude, air pollution, meteorological conditions), and behavioral (e.g., smoking, substance abuse) [7,8,9,10,11,12,13,14,15,16]. Other possible suicide triggers include anxiety or depression, psychiatric illness, mood disorders, and sociocultural factors [17,18,19]. However, risk factors may be different across geographic regions, and risk factors may also change over time. For instance, political violence, an uncommon risk factor for suicide in adolescents, is reportedly associated with suicide among Palestinian youths [20] and periodical economic recession was also found to be associated with elevated suicide rates [21,22].

According to the U.S. Centers for Disease Control and Prevention (U.S. CDC), the age-adjusted average annual suicide rates in the western, southern, mid-western, and northeastern U.S. regions were 14.86, 11.45, 11.52 and 8.97, per 100,000 between 1981 and 1998, respectively. The rates in these four regions were 13.04, 12.37, 11.52 and 8.63 per 100,000 between 1999 and 2015, respectively [23] and the suicide rate in the western region has been significantly higher than its counterparts over both periods of time. One explanation for regional differences in suicide rates is that altitude is much higher in the western region. For instance, Lester (1980) examined state-level suicide rates (1959–1961) and the latitude and longitude of the state capitals in the 48 conterminous states. Lester found that the suicide rates were statistically higher in the western than in the eastern states [24]. Cheng et al. (2005) reported a strong positive correlation between the average suicide rates of counties where the state capitals were located, and mean county altitude (using the altitude or mean altitude of the weather stations in these counties), in the 48 contiguous U.S. states between 1979 and 1998 [25]. Brenner et al. (2011) confirmed a significant positive relationship between county-level suicide rates and the county-center’s altitude, using the suicide rates data between 1979 and 1998 in the 48 contiguous U.S. states [17]. Kim et al. (2011) also analyzed the county-level suicide rates between 1979 and 1998 in the 48 contiguous U.S. states and reported a strong positive association between the average county altitude and both firearm-related and non-firearm-related suicide rates [26]. Moreover, Trgovac et al. (2015) found increased county-level male suicide rates in the western U.S., small areas throughout the Appalachian Region, and the Midwest, using data from 2000 to 2006. In addition, social isolation was theorized as the major cause behind the elevated male suicide risk in high altitude regions [27].

Past studies have also explored several possible pathways connecting suicide rates and altitude. From a social perspective, higher altitude regions may be at a higher suicide risk because these regions tend to be socially and culturally more isolated, and individuals with weak social connections are more likely to view suicide as a solution to personal problems [28,29]. From a social, political and cultural perspective, higher firearm ownership in the western U.S. may partially contribute to higher suicide rates in the region. Firearm suicide is not only the most common, but also the most lethal type of suicide. Since suicidal attempts tend to be impulsive, access to firearms at home will increase the chance of using firearms as a means of suicide [30,31,32]. From a biophysiological and psychological perspective, increased suicide rates could be attributed to depression and/or mood instability, related to high-altitude hypoxia [33,34,35]. However, systematic physiological responses to ambient oxygen levels are extremely complicated and more research is needed to establish a robust causal link between altitude, hypoxia, mood disorder and suicide risk [36,37].

Many previous studies have reported a consistent positive relationship between suicide rates and altitude in the U.S. after adjusting for known independent variables. Such a relationship was also observed elsewhere in the world, including Korea, Taiwan and Ecuador [25,38,39]. However, results in the literature have not always been consistent. For instance, no association was found in a study in Turkey that used suicide rates in 81 provinces in 2007 and 2008 [40]. In a Japanese study, the slope of habitable land was reported to be a more relevant predictor than altitude [41]. A field-based study conducted in the Himalayas and the Andes between 2009 and 2011 disproved the hypothesized link between altitude, depression and suicide, and indirectly rejected the association between suicide and altitude because the prevalence of depression was found to be low in elderly highlanders [42].

Furthermore, two methodological issues in the literature should be noted. First, correlation analysis, an exploratory data analysis tool and ordinary least square (OLS) regression, a global regression method, appeared to be the two most common analytical approaches [17,26,43]. OLS assumes a homogeneous relationship between the outcome variable and predictor variables (e.g., altitude) but a spatially stationary relationship often does not hold for health outcomes [44,45]. Geographically Weighted Regression (GWR), an extension of OLS regression, allows for the exploration of spatial non-stationarity through estimation of local, rather than global, parameters [46]. GWR has been applied extensively in health studies over the past 15 years [47,48,49,50] and has also been adopted in suicide studies [26,51]. However, to the best of our knowledge, GWR has yet to be used to analyze the relationship between suicide and altitude. Second, altitude in the previous studies was represented by a proxy variable such as the location (longitude and latitude) of the state capital [24], the altitude of the state capital city [43], the altitude of the county center [17], or a mean area altitude such as the state mean [52] or the county mean [24]. In addition, altitude was also modeled as an aggregated second-level variable in a multilevel regression model [53]. Among these approaches, the county mean altitude is so far the altitude estimation at the finest geographic scale for an ecological study where the same scale suicide data are also available.

In this study, we investigated the varying association between county-level suicide rates and the mean county altitude, using a more recent U.S. suicide data set (2008 to 2014). The primary objectives of this study were as follows: (1) to compare results from the OLS model and GWR model; (2) to examine whether, and to what extent, the relationship between suicide rates and altitude vary across the 48 contiguous U.S. states, and (3) to explore the association of suicide rates and altitude, after controlling for several county-level demographic, socioeconomic, and behavioral variables. We hope that the findings gleaned from this study can deepen our understanding of the spatial variation of suicide rates and inform policies and initiatives for more precise suicide prevention and intervention.

## 2. Materials and Methods

### 2.1. Suicide Rates

**Figure 1:**
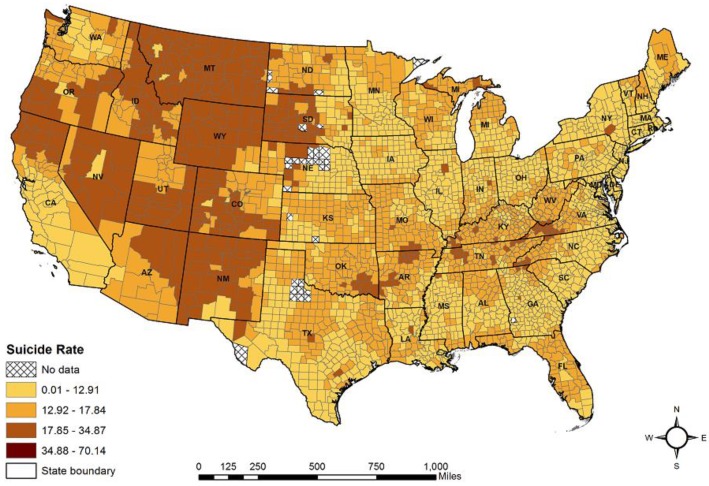
County-level average suicide rates in the 48 contiguous U.S. states between 2008 and 2014 (smoothed and age-adjusted).

The suicide data were collected from the annual mortality files of the National Center for Health Statistics (NCHS) under the U.S. CDC throughout the Web-based Injury Statistics Query and Reporting System (WISQARS) [23,27]. WISQARS provides fatal injury data in all of the 3141 U.S. counties, of which 3102 are in the contiguous U.S. Age-adjusted smoothed suicide rates were downloaded and used in our study. The technical details about the smoothing and age adjusting can be found at WISQARS [23]. In addition, counties with less than 20 cumulative deaths were excluded from our analysis to maintain statistical stability of the data following the U.S. CDC standard [54]. Thus, suicide rates from a total of 3064 counties between 2008 and 2014 were retained and used in our analyses (Figure 1).

### 2.2. Mean County Altitude Data

**Figure 2:**
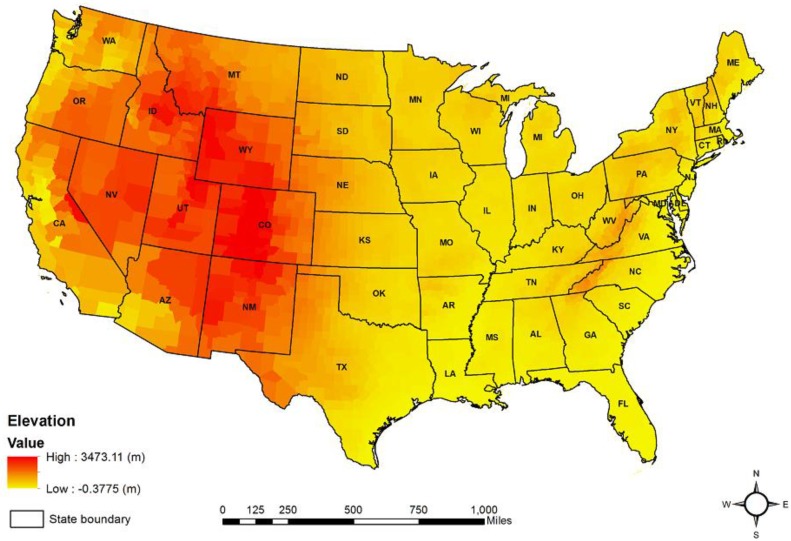
Mean county altitude in the 48 contiguous U.S. states.

A digital altitude model (DEM), with 100-m spatial resolution for the 3064 counties (or equivalent units), was collected from the United States Geologic Survey [55]. The mean altitude of each county was calculated using a zonal statistics operation using the ArcGIS 10.3 software package (Esri, Redlands, CA, United States) [56]. County boundaries were collected from the U.S. Census Bureau [57] (Figure 2).

### 2.3. Independent Variables

We accounted for the effects of potential confounding variables to enhance confidence levels in our analyses [58]. The variables were selected from four different categories based on the literature: (1) health behavior and clinical care variables; (2) social and economic variables; (3) physical environmental variables; and (4) demographic variables, as shown in Table 1 [7,8,9,10,11,12,13,14,15,16]. These county-level variables were collected from various data sources because variables possibly relevant to the suicide rate were not available and not sufficient from NCHS [58]. Moreover, for some predictor variables identified in the literature, such as firearm ownership, religious affiliation, and access to mental health services, county-level data were either unavailable or incomplete, so they were not included in our analyses.

Data of two demographic variables, percentage of the population aged 65 and above, and percentage of the African American population were obtained from the U.S. Census Population Estimates [59]. The health behavioral variable, percentage of the currently smoking population, was collected from the Selected Metropolitan/Micropolitan Area Risk Trends of Behavioral Risk Factor Surveillance System (SMART BRFSS). SMART BRFSS is a premier nationwide system of health-related telephone surveys in the U.S., collecting city- and county-level data about health-related risk behaviors and chronic health conditions [60,61]. In addition, the number of membership associations per 10,000 people (here after the association rate) was obtained from the American Community Survey (ACS) 5-year estimate (2010–2014). Specifically, associations include various membership organizations, such as civic organizations, sports organizations, political organizations, labor organizations, business organizations, and professional organizations. The association rate was used as a proxy indicator to quantify the degree of social support [62]. Finally, four predictor variables, selected from thirteen independent variables, were kept in our final regression models according to the results of statistical testing (Table 2). In addition, there was no multicollinearity between these four variables evaluated using the variance inflation factor (VIF) [63].

### 2.4. Statistical Analyses

First, results from the data skewness analysis indicated that no data transformation was needed. Second, stepwise multiple regression was conducted to select significant predictor variables. Third, both OLS and GWR regression models were constructed to explore both global and local association between suicide rates and altitude, using the four predictor variables. An OLS model can be expressed as

(1) $${\hat{y}}_{i}=b_{0}+\sum_{k}b_{k}x_{ik}^{}+\varepsilon_{i},$$

where ${\hat{y}}_{i}$ is the estimated value of the dependent variable for observation *i*, *b*_0_ is the intercept, *b_k_* is the coefficient for the predictor variable *k*, *x_ik_* is the value of the *k*th predictor variable for observation *i,* and ε*_i_* is the error term [64]. A GWR model, which allows examination of regional variation (spatial non-stationarity) in the relationship between predictor and dependent variables, can be written as

(2) $${\hat{y}}_{i}=b_{0}(u_{i},v_{i})+\sum_{k}b_{k}(u_{i},v_{i})x_{ik}^{}+\varepsilon_{i},$$

where ${\hat{y}}_{i}$ is the estimated value of the dependent variable for observation *i*, $b_{0}(u_{i},v_{i})$ denotes the intercept at the location *i* with coordinates $\left(u_{i},v_{i}\right)$; $b_{k}(u_{i},v_{i})$ represents the coefficient estimate for the predictor variable *k* at location *i* with coordinates of $\left(u_{i},v_{i}\right)$; $x_{ik}$ denotes the observation on independent variable *k* at location *i*, and $\varepsilon_{i}$ is the error term [64]. Instead of solving one single regression equation, GWR calibrates a separate regression equation for each observation, assuming that closer observations have greater influence on the estimation of the regression parameters than those further away ones [46]. The weight given to each observation is determined by a distance decay function.

Raster-based coefficient surfaces are then created using estimated coefficients at all the sample locations. A weight matrix is constructed for each raster cell, relating the location of the cell to the locations of all the other observations in the dataset. The weighting matrix is built based on a distance decay function, which is dependent on a chosen bandwidth. Two methods are commonly used to select the bandwidth: the cross-validation (CV) method and the Akaike Information Criteria (AIC) method [64]. Specifically, CV seeks the bandwidth that minimizes the CV score, expressed as

(3) $$\mathrm{CV}=\sum_{i=1}^{n}(y_{i}-{\hat{y}}_{\neq i}{)}^{2},$$

where *n* represents the number of observations. Note that observation *i* is excluded from the calibration when predicting the value at location *i* [63,64]. Alternatively, the AIC method finds the bandwidth that minimizes the AIC score, expressed as

(4) $$\mathrm{AIC}=2n\log_{\varepsilon }(\hat{\sigma })+n\log_{\varepsilon }(2\pi )+n\{\frac{n+tr(S)}{n-2-tr(S)}\},$$

where *tr*(*S*) represents the trace of the hat matrix. The hat matrix describes the relationship between the fitted values and the observed values. The diagonal elements of the hat matrix specify the influence of each observed value on each fitted value for the same observation [65]. The AIC method has an advantage over the CV method in that it considers the degrees of freedom, which may vary between models centered on different observations [64]. In addition, bandwidth can either be fixed or adaptive. The fixed function uses an optimal bandwidth for the entire study area and all observations that fall within it are used in the subset regression. In contrast, the adaptive function allows bandwidth to vary based on the density of observation points [27,64,66]. Given the uneven distribution of counties in the data sets, the adaptive bandwidth with the AIC optimization method was applied in our analysis. 

Moreover, since a GWR model provides separate parameter estimate measures, goodness-of-fit, and significance assessment for every observation in the dataset, regional variation (spatial non-stationarity) and significance of the association between the dependent variable and predictor variables, can be visualized and interpreted using maps made using the estimated values of the above measures [46].

## 3. Results

Table 3 shows the mean, standard deviations (SD), and range statistics of both dependent and independent variables. There was a total of 263,210 suicide deaths in the 3064 contiguous U.S. counties (or parishes) from 2008 to 2014. The seven-year smoothed age-adjusted average suicide rate per 100,000 population was 13.53 with a range from 5.17 to 70.14 and a SD of 3.53. The mean county altitude ranged between −0.37 m (above sea level) and 3473.11 m with a mean of 438.42 m and a SD of 509.71 m. The other four predictor variables all showed different degrees of variation across the states. On average, 18.39% of a county’s population smoked with a SD of 3.71%. The African American population, on average, accounted for 12.39% of the county population with a SD of 14.33%. The proportion of a county’s population aged 65 and above was, on average, 17.58% with a SD of 4.3%. The mean county association rate was 13.83 membership associations per 100,000 population with a SD of 6.78. The variations in these predictor variables indicate that suicide incidences occurred in regions with heterogeneous behavioral, socio-demographic, and environmental characteristics.

Figure 1 shows the suicide rates (smoothed and age-adjusted) in the contiguous U.S. counties from 2008 to 2014 using natural break classification [20]. Overall, suicide rates were much higher in the western counties compared to those in the other regions. In addition, the global Moran’s *I* test (Moran’s *I* = 0.447; z = 87.94; *p* < 0.001) suggested a spatially dependent county-level suicide pattern during our study period.

**Figure 3:**
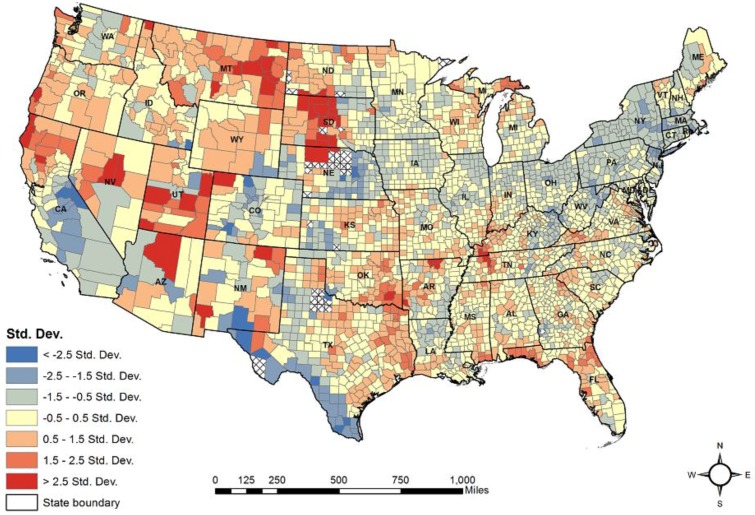
The residuals of the OLS model.

Table 4 summarizes the results from the OLS regression model. A R^2^ value of 48.3% indicates that the five predictor variables explained almost half of the total variance in the county-level suicide rates. The VIF was less than two for all predictor variables, indicating no multicollinearity in the data. The suicide rates were significantly (*p* < 0.001) related to all five of the predictor variables. A positive relationship was found with the altitude, percentage of smoking population, and percentage of population aged 65 and above, while a negative relationship was discovered with the percentage of African American population and the association rate. Moreover, every 100 m increase in mean county altitude led to an increase in suicide rates of 0.4/100,000 (*B* = 0.004; *p* = 0.000), after adjusting the other four predictor variables. In addition, the model residuals were spatially autocorrelated (Moran’s *I* = 0.323; z = 63.526; *p* < 0.001), meaning that the county-level suicide rates were spatially dependent across the 48 U.S. states. The residuals also indicated that suicide rates were underestimated primarily in the western states including Utah, Wyoming, Montana, South Dakota, and North Dakota (Figure 3).

**Figure 4:**
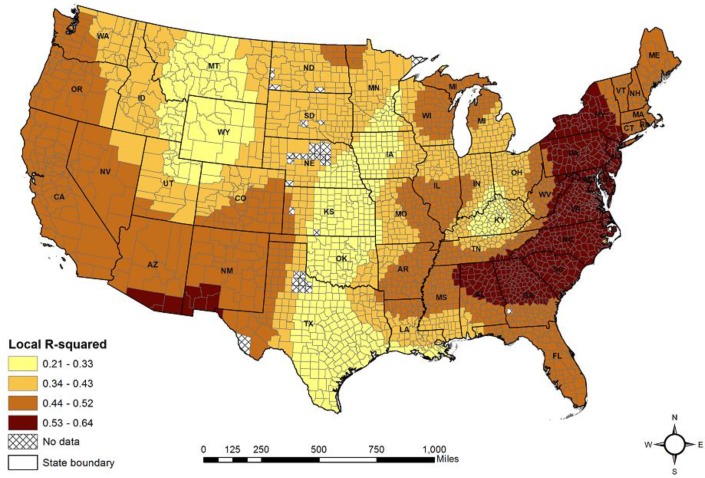
Local *R*^2^ values from the GWR model.

Table 5 presents the model fit measure along with the ranges of local coefficient estimates for the predictor variables from the GWR model. Compared with the results from the OLS model, the GWR model fit the data better with a higher R^2^ value (average: 0.62; range: 0.21–0.64, Figure 4) and a lower AIC value (13,593.68). In addition, the spatial autocorrelation in the GWR model was significantly reduced, though was not totally eliminated, as indicated by the Moran’s *I* statistic (Moran’s *I* = 0.171; z = 33.656; *p* < 0.001).

**Figure 5:**
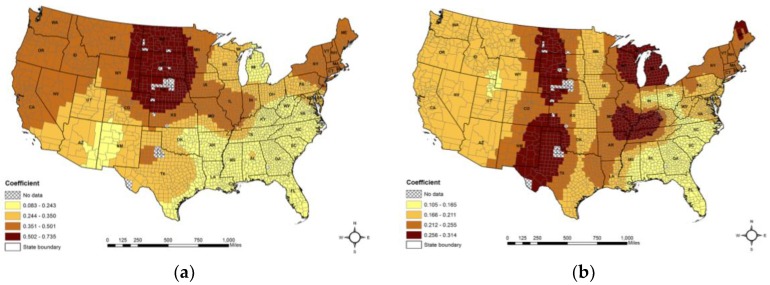

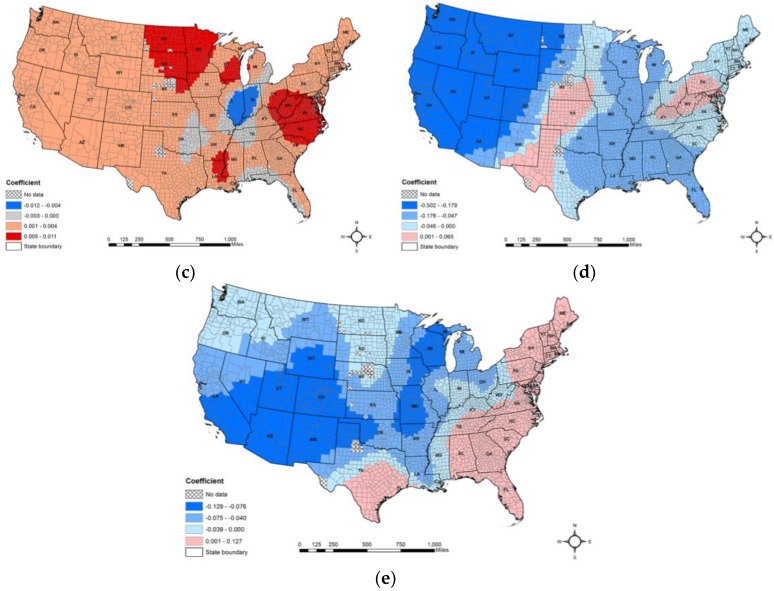
Coefficient estimates of the GWR model: (**a**) percentage of the local population that smoke; (**b**) percentage of the population aged 65 and above; (**c**) mean county altitude; (**d**) percentage of the African American population; and (**e**) the association rate.

Figure 5 shows the spatial patterns of the estimated coefficients of the five predictor variables. For smoking, stronger positive relationships were found in the northern plains, the Midwest, and northeast, particularly in portions of North Dakota, South Dakota, and Nebraska (Figure 5a). For percentage of the population aged 65 and above, stronger positive relationships could be seen in parts of the Midwest (North Dakota, South Dakota, and Nebraska), the southwest (Kansas, Oklahoma, New Mexico, and Texas), the northcentral (Michigan and eastern parts of Wisconsin), and portions of the central (Missouri, Illinois, Kentucky, and Tennessee) states (Figure 5b).

For mean county altitude, stronger positive relationships can be seen in portions of the northern and northern plains regions (Minnesota, North Dakota, and South Dakota), southeastern regions (West Virginia, Virginia, and North Carolina), and Louisiana. Stronger negative relationships were found in portions of Indiana and Illinois (Figure 5c). For the percentage of the African American population, stronger negative relationships appeared primarily in western states and positive relationships were discovered in the southwest (Texas, Kansas, and Nebraska) and the northeast (Kentucky, Ohio, Virginia, and West Virginia, and Pennsylvania) states (Figure 5d). For the association rate, stronger negative relationships were found in portions of the southwest, the north and the central north states, and positive relationships were seen in south Texas and all eastern states (Figure 5e).

**Figure 6:**
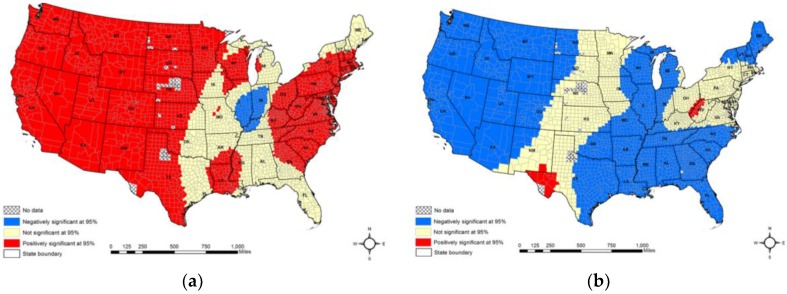

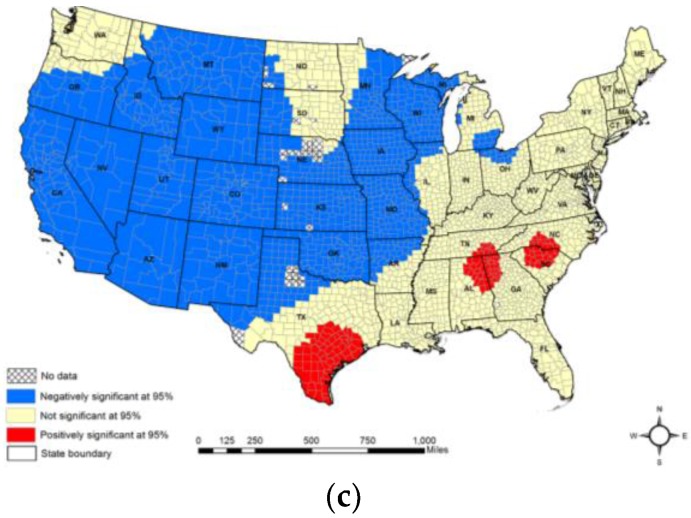
Significance map of the GWR model; (**a**) mean county altitude; (**b**) percentage of the African American population; and (**c**) the association rate.

Level of significance of the coefficients also varied across the 48 U.S. states. While consistent positive relationships were seen with smoking and elderly population, both negative and positive relationships were found with mean county altitude, percentage of the African American population, and the association rate. Moreover, mean county altitude had more positive relationships than the negative ones (64.56 percent vs. 4.56 percent) (Figure 6a). A negative relationship was found in 61.95 percent of the counties with of the percentage of the African American population (Figure 6b) and 37.74 percent of the counties with the association rate (Figure 6c). In addition, a positive relationship was found in 1.30 percent of the counties with the percentage of African American population (Figure 6b) and 4.99 percent of the counties with the association rate (Figure 6c).

## 4. Discussion

Our study expanded the existing literature on the association between suicide and altitude. A more recent U.S. suicide data set (2008–2014) was analyzed using both global and localized regression (OLS and GWR) models, and the mean county altitude was estimated with the best possible spatial resolution to our knowledge. Results from our GWR model showed that there existed a significant positive relationship between the county-level suicide rates and the county mean altitude, in close to two-thirds of the contiguous U.S. counties (particularly in the western and northeastern regions), after controlling for four predictor variables (i.e., percentage of the smoking population, percentage of population aged 65 and over, percentage of African American population African American, and the association rate). Our findings were generally consistent with the findings in the previous U.S. studies. However, the magnitude of the effect of altitude of our estimation was smaller than those in the literature [17,26,38]. Moreover, both unemployment and rurality were found to be insignificant in our models (Table 2) but were significant in most of the past studies [12,26,67,68].

In our study, we included all counties in our models so that continuous surfaces could be generated using the results from the GWR model to visualize the spatial variation of the relationship between the suicide rates and altitude across the contiguous U.S. Some scholars, concerned about the impacts of the floor effect [17], included only counties above a certain threshold altitude in their analyses. For instance, Huber et al. (2014) excluded counties with an altitude lower than 308.4 m (1000 ft.) from their models. As the floor effect may impact our modeling results, readers should interpret our modeling results with caution [53].

Our GWR model also identified areas where relationships between suicide rates and predictor variables were contradictory to most of the other counties. For instance, a pocket of counties in Illinois and Indiana showed a significant negative relationship between suicide and altitude. Some counties in northwest Texas and Ohio and West Virginia showed a significant positive relationship between suicide and the percentage of the American-African population. Significant positive relationships between suicide and the association rate were found in some areas in southeast Texas, north Alabama, south of North Carolina, and north of South Carolina. Several explanations are available to justify such relationships contradictory to mainstream theories as well as and the dominating patterns. Effects in these areas might be counteracted by local mechanisms that were not captured by the current model. For instance, a negative relationship between suicide and altitude might be related to the floor effect of the altitude in the area. One major advantage of using the GWR model is to identify regions with “abnormal” relationships so that more thorough research may be conducted in these areas.

Several integration mechanisms have been developed to explain the association between suicide rates and altitude. First, suicide is closely related to social relationships, social integration, and social regulation [28]. Thus, suicide occurs more frequently among those with weaker social connections and inadequate social support. A large portion of the population in the western U.S. are at a higher suicide risk because they reside at higher altitude regions that are much less socially connected. Second, it is well recognized that firearm ownership has been closely related to firearm suicide rates in the U.S. and thus, higher firearm ownership in the western U.S. may be a significant contributing factor for the higher suicide rates [30,31,32]. Third, from a biophysiological perspective, high altitude hypoxia may worsen mood, especially to those emotionally unstable, which may result in an increased suicide risk [58]. However, the relationship between altitude, hypoxia, mood and suicide needs further investigation. Some researchers argue that there lacks clinically proven evidence to substantiate a pathway connecting living in high altitude regions, hypoxia, worsening mood, depression, and suicide [69,70]. Previous studies also found that the relationship between high altitude and depression may be compensated by other factors. For instance, Ishikawa et al. (2013) found that the prevalence of depression was low in elderly highlanders living in the Himalayas and the Andes. The low prevalence was attributed to a deep devotion to religion and tight interpersonal networks [42]. In short, despite recent progress, robust pathways between altitude and suicide have yet to be established.

There are some limitations in this study. First, the reliability of the suicide data is questionable due to the well-known systematic under-reporting of suicide incidence [27,71]. Second, certain aspects of the GWR method, particularly regarding to the multicollinearity issue and approaches to calculating goodness of fit statistics, are still open to debate. For instance, some researchers contended that GWR might induce localized collinearity due to the inability of GWR to consistently differentiate the spatially stationary and non-stationary generating processes [72,73]. Fotheringham and Oshan [74], on the other hand, demonstrated that with controlled simulation GWR is sufficiently robust to withstand the multicollinearity effects [74]. For these reasons, GWR should be treated as more an exploratory data analysis tool to investigate the spatial non-stationarity relationship. Third, like most of the previous analyses, our study is threatened by ecological fallacy. Our data were aggregated at the county level, and individual-level risk factors were masked in our models. For example, residential history of individuals were not available so the altitude level an individual was residing at the time of the suicide was unknown, thus, the link between suicide and altitude is not robust. Lastly, other significant predictor variables including firearm ownership, religious affiliation, and accessibility of mental health services were not adjusted by our models because county-level data were not available when this study was conducted.

Furthermore, existing studies including ours have focused primarily on the spatial variations of suicide rates, but the temporal dimension of a spatial problem is at least equally important [75,76,77]. Geographical and temporal weighted regression (GTWR), an extension of GWR, has already been proposed to model the spatiotemporal pattern of local nonstationary processes [77,78]. In addition, past studies have suggested that specific meteorological conditions, such as atmospheric pressure, temperature, wind velocities, and hours of sunlight, might also be associated with suicide risk. However, the possible interaction between these factors and altitude was not considered in our analyses [79,80]. Finally, most of the previous studies pooled suicide data over several years to mitigate the small area problem. The assumption of this treatment of the raw data is that suicide is independent of time. However, if this assumption fails to hold, then the estimation of the suicide risk may be biased and spatial models for repeated time periods [81,82] and/or space-time models [83,84,85] are possible solutions for this problem.

## 5. Conclusions

We in this study examined the spatially varying relationship between county-level suicide rates and altitude using the most recent suicide data that are available in the U.S. Results from our regression models confirmed an overall positive relationship between county-level smoothed age-adjusted suicide rates and mean county altitude, after controlling for four predictor variables including smoking, age of population, percentage of African American population, and the association rate. The relationships varied considerably across the contiguous U.S. Counties in the western and northeastern regions appeared to mostly align well with the hypothesized positive relationship between altitude and suicide risk, although a pocket of counties in Illinois and Indiana showed a reversed relationship. Future research may further investigate areas with a negative relationship as well as testing additional predictor variables, such as religious affiliation and accessibility of mental health facilities and services. Future research may also simultaneously model the varying relationship in space and over time. We hope that a better understanding of the association between suicide and altitude may help identify high-risk regions for further research and/or for formulating targeted prevention and intervention strategies.

## Figures and Tables

**Table 1:** List of independent variables.

Health behavior and clinical care variables
Percentage of the population who smoke Percentage of the population who are obese Ratio of primary care physicians to population
Social and economic variables
Percentage of adults aged 25–44 years with some post-secondary education Percentage of population aged 16 and older who are unemployed but seeking work Percentage of children that live in a single-parent household Number of membership associations per 10,000 population
Physical environmental variables
Percentage of households with at least one of the following four housing problems: overcrowding, high housing costs, lack of kitchen or lack of plumbing facilities Mean county altitude in meters
Demographic variables
Percentage of individuals aged 65 and over Percentage of African American Percentage of females Percentage of rural areas

**Table 2:** OLS regression analyses of all independent variables for suicide rates in the NCHS.

Model—With Mean County Altitude and Independent Variables—*R*-Squared = 0.485				
	Coefficient	S.E	*t*-Value	*p*-Value
Intercept	5.147	1.270	4.053	0.000 ^a^
% smokers	0.312	0.019	16.556	0.000 ^a^
% obesity	0.005	0.017	0.298	0.766
Primary care physician rate	0.002	0.002	1.381	0.167
% college education	0.006	0.006	1.101	0.271
% unemployment	0.008	0.029	0.292	0.771
% of single-parent households	0.006	0.007	0.825	0.410
Association rate	−0.053	0.009	−6.142	0.000 ^a^
% severe housing problems	0.013	0.014	−0.913	0.361
% aged 65 and over	0.226	0.015	15.566	0.000 ^a^
% African American	−0.036	0.005	−7.092	0.000 ^a^
% female	−0.057	0.024	−2.318	0.021
% rural area	0.002	0.002	0.865	0.387
Mean county altitude	0.004	0.000	35.974	0.000 ^a^

^a^ Significant at *p* < 0.001.

**Table 3:** Descriptive statistics for dependent and independent variables for NCHS.

	Mean	SD	Range
Suicide rates (per 100,000 population)	13.53	3.53	5.17–70.14
% smokers	18.39	3.71	6.90–41.20
% obesity	30.94	4.46	10.70–46.60
Primary care physician rate	55.74	34.75	0–469.23
% college education	56.27	11.65	2.70–100
% unemployment	6.26	2.30	1.20–23.70
% single-parent households	32.47	10.32	0–100
Association rate	13.83	6.78	0–81.30
% severe housing problems	14.47	4.86	2.18–71.26
% aged 65 and over	17.58	4.36	4.10–52.90
% African American	12.39	14.33	0–84.90
Association rate	13.83	6.78	0–81.30
% female	49.91	2.27	30.10–56.80
% rural area	58.82	31.50	0–100
Mean county altitude (m)	438.42	509.71	−0.37–3473.11

**Table 4:** Partial results from the OLS regression model

Model—With Mean County Altitude and Potential Covariates—*R*-Squared = 0.483, AIC Value: 14,432.13				
	Coefficient	S.E	*t*-Value	*p*-Value
Intercept	2.730	0.336	8.134	0.000 ^a^
Mean county altitude	0.004	0.000	40.691	0.000 ^a^
% Smokers	0.334	0.013	25.196	0.000 ^a^
% aged 65 and over	0.219	0.012	18.828	0.000 ^a^
% African American	−0.036	0.004	−10.093	0.000 ^a^
Association rate	−0.0045	0.007	−6.028	0.000 ^a^

^a^ Significant at *p* < 0.001.

**Table 5:** Partial results from the GWR model.

Model—With Mean County Altitude and Four Predictor Variables—R-Squared: 0.620, AIC Value: 13,593.68					
	Coefficient Range		Percentage of Counties by 95% of *t* Statistic		
	Min.	Max.	*t* ≤ −1.96	−1.96 < *t* < 1.96	*t* ≥ 1.96
Intercept	−5.68	10.41	6.62	16.75	76.63
Mean county altitude	−0.01	0.01	4.56	30.88	64.56
% Smokers	0.08	0.74	0.04	0	99.96
% 65 and over	0.11	0.31	0	0	100.00
% African American	−0.50	0.07	61.95	36.75	1.30
Association rate	−0.13	0.13	37.74	57.27	4.99
